# Improving Meat Quality, Safety and Sustainability in Monogastric Livestock with Algae Feed Additives

**DOI:** 10.3390/foods14061007

**Published:** 2025-03-16

**Authors:** José A. M. Prates

**Affiliations:** 1 CIISA—Centro de Investigação Interdisciplinar em Sanidade Animal, Faculdade de Medicina Veterinária, Universidade de Lisboa, Av. da Universidade Técnica, 1300-477 Lisboa, Portugal; japrates@fmv.ulisboa.pt; 2 Associate Laboratory for Animal and Veterinary Sciences (AL4AnimalS), Av. da Universidade Técnica, 1300-477 Lisboa, Portugal

**Keywords:** microalgae, seaweeds, monogastric animals, meat quality, meat safety, meat sustainability

## Abstract

Integrating algae (microalgae and seaweeds) into monogastric animal diets presents significant opportunities to improve meat quality, safety, and sustainability. This review synthesizes current knowledge on the nutritional and bioactive compounds found in key microalgae (e.g., *Chlorella vulgaris*, Spirulina, and *Nannochloropsis*) and seaweeds (e.g., *Ascophyllum nodosum*, *Ulva*), emphasizing their potential benefits for animal health and meat production. Algae-enriched diets substantially increase meat omega-3 fatty acid content and antioxidant capacity, thereby enhancing nutritional value, sensory appeal, and shelf life by mitigating lipid and protein oxidation during storage. Additionally, bioactive compounds in algae demonstrate potent antimicrobial activities capable of reducing pathogenic bacteria such as *Salmonella*, *Escherichia coli*, and *Campylobacter*, significantly contributing to improved meat safety. Environmentally, algae cultivation reduces dependency on arable land and freshwater, promotes nutrient recycling through wastewater use, and substantially decreases greenhouse gas emissions compared to traditional livestock feeds. Nevertheless, challenges persist, including high production costs, scalability concerns, variability in nutrient composition, potential contamination with heavy metals and other toxins, and regulatory constraints. Overcoming these limitations through advancements in cultivation technologies, optimized inclusion strategies, and comprehensive market and regulatory analyses is essential to fully realize the potential of algae in sustainable monogastric livestock feeding systems.

## 1. Introduction

Ensuring meat quality, safety and sustainability is a priority in monogastric livestock production, particularly in poultry and swine, which dominate global meat consumption [1]. These sectors are critical for meeting the increasing demand for animal protein. However, they face significant challenges related to production practices, environmental impacts and consumer health concerns [2]. Addressing these issues requires innovative strategies to improve meat quality and safety while promoting sustainability in production systems.

Meat quality encompasses attributes such as nutritional value, sensory appeal, and shelf life, essential for consumer acceptance and marketability. Meat safety refers to the absence of harmful pathogens, chemical residues, and contaminants, ensuring that meat products pose no health risks. Sustainability focuses on minimizing the environmental impact of production, including greenhouse gas emissions and nutrient pollution, while maintaining economic viability [3]. In monogastric production, poultry and pork account for more than 70% of global meat consumption [1]. However, the large scale of these industries has amplified concerns about environmental degradation, foodborne illnesses, and the overreliance on synthetic additives, prompting a demand for sustainable and natural alternatives.

Traditional feed additives, such as synthetic antioxidants and antibiotics, have long been employed to enhance meat quality and safety [4]. Synthetic antioxidants effectively prevent lipid oxidation, preserving sensory attributes and extending product shelf life. However, growing concerns regarding their potential toxicological effects and environmental persistence have prompted the exploration of natural alternatives [5]. Recent research has highlighted the efficacy of natural antioxidants such as rosemary extracts [6], grape seed polyphenols [7], and algae-derived compounds, including carotenoids and polyphenols from species like *Chlorella vulgaris* [8] and *Ascophyllum nodosum*, which significantly reduce lipid oxidation, improve meat’s sensory qualities, and extend shelf life, offering safer and more sustainable solutions compared to synthetic antioxidants. Similarly, antibiotics have been widely used as growth promoters and therapeutic agents. Although effective in the short term, this practice has contributed to the rise of antimicrobial resistance, a global health crisis where resistant bacteria can transfer to humans through the food chain, posing severe health risks [9,10,11]. Regulatory restrictions on antibiotic use, particularly in the European Union, have intensified the need for effective and sustainable alternatives.

In this context, algae (microalgae and seaweeds) have emerged as promising, natural feed additives for monogastric livestock. These marine-derived organisms are rich in bioactive compounds, including polyunsaturated fatty acids (PUFAs), antioxidants, and prebiotic polysaccharides, which offer multiple benefits to animal health and meat production [8,12,13,14]. Notable species such as Spirulina (*Limnospira platensis*), *Chlorella vulgaris* and *Ascophyllum nodosum* possess nutrient-dense profiles containing essential amino acids, vitamins, minerals, and bioactive compounds. Combining microalgae and seaweeds in monogastric feeding is particularly advantageous due to their complementary nutritional profiles, antimicrobial properties, and sustainability benefits. Microalgae provide high levels of proteins and omega-3 fatty acids, while seaweeds contribute polysaccharides, antioxidants and minerals. Together, they offer a holistic approach to improving meat quality, safety, and environmental impact, addressing the challenges associated with traditional feed additives. Furthermore, these organisms can be cultivated on nonarable land with minimal freshwater requirements, enhancing their status as an environmentally sustainable option [15,16].

Research highlights several advantages of incorporating microalgae and seaweeds into monogastric diets. These include enhancing meat nutritional quality by increasing the deposition of omega-3 fatty acids and other beneficial compounds, reducing lipid oxidation and improving shelf life due to potent antioxidant properties, and decreasing the prevalence of foodborne pathogens through antimicrobial effects [2,8]. Additionally, their use supports sustainability by reducing greenhouse gas emissions, nutrient runoff, and reliance on traditional feed resources.

This review evaluates the potential of microalgae and seaweeds, single or blended, as feed additives for monogastric livestock, focusing on their impact on meat quality, safety, and sustainability. To support this evaluation, a comprehensive search was conducted across databases such as Google Scholar, PubMed, Scopus and Web of Science using targeted terms like “microalgae,” “seaweeds,” “monogastric diets,” “meat quality,” “meat safety,” and “meat sustainability,” with priority given to recent (less than 10 years) studies. The review examines the nutritional and bioactive properties of these marine resources, their effects on animal performance, gut health, meat safety and shelf life, as well as their environmental benefits. It also addresses challenges such as economic feasibility and regulatory barriers. By synthesizing current knowledge, this review highlights the practical applications of microalgae and seaweeds in sustainable monogastric feeding strategies and identifies opportunities for future research and innovation.

## 2. Brief Description of Nutritional and Bioactive Composition of Algae

Algae are valuable feed additives for monogastric animal diets due to their rich nutritional profiles and diverse bioactive compounds. Microalgae are unicellular, photosynthetic microorganisms found in freshwater and marine environments, while seaweeds (macroalgae) are larger, multicellular marine algae classified into brown, red, and green varieties. Together, these organisms provide a wide range of proteins, lipids, antioxidants, and polysaccharides, which can improve animal health, meat quality, and sustainability.

### 2.1. Nutritional and Bioactive Composition of Microalgae

Microalgae offer high levels of proteins, omega-3 fatty acids, and a diverse range of bioactive compounds beneficial for livestock health and meat quality. Spirulina is particularly recognized for its exceptionally high protein content, which can reach up to 70% of its biomass [13]. Additionally, Spirulina is rich in gamma-linolenic acid (GLA), a PUFA, and contains several valuable bioactive components, including β-carotene, phycobiliproteins, vitamins, and antioxidant peptides, which collectively enhance antioxidant capacity and immune response. *Chlorella vulgaris*, containing 50–60% protein, is another prominent microalga and a significant source of alpha-linolenic acid (ALA), an essential omega-3 fatty acid [12]. Beyond these nutrients, *Chlorella* provides a variety of bioactive substances, such as vitamins, essential amino acids, carotenoids, polysaccharides, and peptides, contributing to its broad antioxidant and immunomodulatory properties. *Nannochloropsis* species are notable for their high concentrations of long-chain omega-3 PUFAs, particularly eicosapentaenoic acid (EPA) and docosahexaenoic acid (DHA) [17]. *Schizochytrium* species are also rich sources of these omega-3 fatty acids, particularly DHA. *Schizochytrium*-derived oils are increasingly used as sustainable alternatives to fish oil in both human and animal nutrition, providing a plant-based source of high-quality omega-3 [18]. These omega-3 fatty acids are essential for improving the nutritional quality of meat and supporting anti-inflammatory processes and overall animal health [16]. Additionally, microalgae produce other bioactive molecules, such as polyphenols, sterols, and pigments that further enrich their nutritional and functional profiles [19]. Overall, the combination of proteins, omega-3 fatty acids, antioxidants, and various other bioactive compounds makes microalgae promising candidates for enhancing animal diets and improving meat quality.

### 2.2. Nutritional and Bioactive Composition of Seaweeds

Seaweeds, classified into brown, red, and green types, offer diverse nutritional and bioactive components beneficial for animal health and meat quality. Brown seaweeds such as *Ascophyllum nodosum* and *Laminaria digitata* are notably rich in fucoidan, alginate, and polyphenols, contributing to improved gut health, growth performance, and reduced oxidative stress [8,20]. Red seaweeds, including *Gracilaria*, *Porphyra*, and *Palmaria palmata*, contain substantial protein levels (up to 18%), dietary fiber, essential minerals (calcium, phosphorus, iron), and bioactive polysaccharides such as agar and carrageenan which support gut microbiota, immune function, and overall animal performance [21]. Green seaweeds, exemplified by *Ulva* and *Enteromorpha*, provide valuable polysaccharides like ulvan, known for prebiotic and immunomodulatory effects, as well as phenolic compounds offering antioxidant properties [22]. By encompassing a broader range of seaweed species, this comprehensive nutritional and bioactive profile highlights their potential in enhancing gut health, animal performance, and meat product quality.

### 2.3. Bioactive Compounds and Their Benefits

The benefits of microalgae and seaweeds are derived from their diverse array of bioactive compounds that contribute to meat quality, safety, and animal health. Omega-3 fatty acids, such as EPA and DHA, found in *Nannochloropsis* and *Chlorella vulgaris*, enhance meat quality by increasing omega-3 content and reducing inflammation [23]. Carotenoids, including β-carotene and phycobilins in *Chlorella* and Spirulina, provide potent antioxidant and immune-boosting effects, helping improve meat quality and shelf life [24]. Polyphenols found in seaweeds like *Ascophyllum nodosum* improve oxidative stability and extend meat shelf life by reducing lipid oxidation [25]. Additionally, chlorophylls present in many microalgae aid in detoxification and exhibit antioxidant activity, supporting overall animal health [26]. Polysaccharides such as fucoidan, laminarin and ulvan, found in brown and green seaweeds, exhibit prebiotic, immunomodulatory and antimicrobial properties, promoting gut health and reducing harmful pathogens [27].

### 2.4. Synergies and Practical Applications

The combination of microalgae and seaweeds in monogastric feeding offers synergistic benefits due to their complementary nutritional and bioactive profiles. Microalgae provide high-quality proteins, essential omega-3 fatty acids, vitamins, and various bioactive compounds such as antioxidants and peptides, while seaweeds contribute valuable polysaccharides, antioxidants, polyphenols, and essential minerals. Together, these marine resources significantly enhance animal health, meat nutritional quality, and oxidative stability, thus improving product shelf life and safety. Moreover, their cultivation on non-arable land with minimal freshwater usage highlights their potential as sustainable alternatives to conventional feed sources. Integrating microalgae and seaweeds into monogastric diets can address environmental sustainability concerns, reduce reliance on traditional feed additives, and support the economic viability of livestock production. Consequently, the blended use of microalgae and seaweeds holds considerable promise for enhancing monogastric feeding strategies, a concept recently supported by applications in aquafeeds [28].

## 3. Enhancing Meat Quality Through Algae

Integrating algae into monogastric animal diets offers substantial benefits for enhancing meat quality through nutritional improvements, sensory attributes, and shelf-life extension. These marine-derived feeds are rich in omega-3 fatty acids, antioxidants and other bioactive compounds that contribute to healthier, more appealing, and longer-lasting meat products.

### 3.1. Nutritional Improvements

Microalgae and seaweeds significantly improve the nutritional profile of meat by increasing the levels of beneficial compounds, particularly omega-3 PUFA and antioxidants.

Microalgae such as *Nannochloropsis* and *Chlorella vulgaris* are excellent sources of EPA and DHA. When included in animal diets, these microalgae increase the deposition of omega-3 fatty acids in muscle tissues. Studies have shown that poultry-fed diets enriched with microalgae exhibit significantly higher levels of omega-3 fatty acids in their meat, improving its nutritional profile and providing cardiovascular health benefits for consumers [29,30]. Omega-3-enriched meat has been associated with reduced inflammation, improved heart health, and better cognitive function in humans [31].

Seaweeds like *Ascophyllum nodosum* and *Gracilaria* are rich in polyphenols, carotenoids, and essential minerals. When included in animal feed, these bioactive compounds are deposited in muscle tissues, enhancing the meat’s antioxidant capacity and overall nutritional value [20]. This deposition helps protect meat against oxidative damage, thereby improving its health benefits and supporting animal well-being [8,32]. By incorporating these marine-derived additives, producers can deliver meat products with enhanced levels of antioxidants, which are essential for reducing oxidative stress and supporting immune function.

### 3.2. Sensory Attributes

Microalgae and seaweeds contribute to improving the color stability, flavor, and texture of meat, enhancing its sensory appeal and consumer acceptance.

Antioxidants present in microalgae and seaweeds, such as carotenoids, chlorophylls, and polyphenols, help maintain meat color by preventing lipid oxidation and myoglobin degradation. Diets supplemented with Spirulina and *Chlorella vulgaris* have been shown to improve meat redness and overall color uniformity, which is crucial for consumer appeal [33,34]. The antioxidant activity delays the oxidation of myoglobin, preserving the bright red color associated with fresh meat and extending its visual appeal during storage.

The inclusion of microalgae and seaweeds at moderate levels (1–3%) can enhance the flavor profile and texture of meat. The deposition of omega-3 fatty acids and bioactive compounds in muscle tissues can lead to a richer, more complex flavor and improved tenderness. However, high inclusion rates may introduce undesirable flavors, described as “fishy” or “seaweed-like,” due to the presence of marine-derived lipids and minerals [35,36]. Therefore, optimizing the inclusion rate is essential to balance the nutritional benefits with desirable sensory properties.

### 3.3. Shelf-Life Improvements

The antioxidant properties of microalgae and seaweeds play a critical role in extending the shelf life of meat by reducing lipid and protein oxidation during storage.

Polyphenols and carotenoids from seaweeds like *Ascophyllum nodosum* and *Ulva* inhibit lipid and protein oxidation, maintaining the quality of meat over time. Studies have demonstrated that meat from animals fed diets enriched with algae show significantly lower levels of malondialdehyde (MDA), a key marker of lipid oxidation, compared to conventional meat [8,33]. This reduction in oxidation helps retain the meat’s flavor, color, and texture during storage, extending its marketable life and reducing food waste. Improved oxidative stability also reduces the formation of off-flavors, ensuring that meat maintains its sensory qualities for a longer period.

### 3.4. Synergistic Effects for Comprehensive Meat Quality Enhancement

Combining microalgae and seaweeds provides a synergistic approach to enhancing meat quality. Microalgae contribute high-quality proteins and omega-3 fatty acids, while seaweeds provide antioxidants and polysaccharides that support oxidative stability and gut health. Together, these feed additives offer a balanced strategy to improve the nutritional profile, sensory appeal, and shelf life of meat. This comprehensive approach addresses consumer demand for healthier, safer, and more sustainable meat products.

By integrating microalgae and seaweeds into monogastric diets, producers can meet market demands for nutritious and high-quality meat while promoting environmentally sustainable production practices. Continued research and optimization of inclusion rates will help maximize these benefits and ensure consistent meat quality [30,37].

Table 1 summarizes the nutrients and bioactive compounds present in the most relevant microalgae and seaweeds and their impact on specific meat quality parameters.

## 4. Improving Meat Safety with Algae

The incorporation of algae, including microalgae and seaweeds, into monogastric feeding strategies offers substantial benefits for meat safety. Through antimicrobial, antioxidant, detoxification, and immunomodulatory mechanisms, algae-derived bioactive compounds provide natural, effective, and sustainable means to enhance the safety and quality of meat products.

### 4.1. Antimicrobial Properties

Microalgae and seaweeds exhibit robust antimicrobial properties effective against key foodborne pathogens such as *Salmonella*, *Campylobacter*, and *Escherichia coli*. Seaweed species, including *Ascophyllum nodosum*, *Laminaria digitata*, and *Fucus vesiculosus* contain bioactive compounds such as phlorotannins, polysaccharides, and halogenated metabolites, which demonstrate broad-spectrum antimicrobial activity. Fucoidans and phlorotannins disrupt bacterial cell membranes, reduce intracellular ATP, and decrease bacterial adherence to gut epithelia, effectively controlling pathogens like *Salmonella agona*, *Campylobacter jejuni*, *Escherichia coli*, and *Streptococcus suis* [27,40]. These actions significantly lower pathogen loads in animals, directly reducing contamination risks in meat products.

### 4.2. Gut Health and Microbiota Modulation

Algal bioactive compounds enhance gut health by promoting beneficial microbiota and suppressing pathogenic bacteria, directly influencing meat safety. Seaweed-derived polysaccharides, particularly laminarin and fucoidan, function as prebiotics, selectively enhancing the growth of beneficial gut microbiota such as *Lactobacillus* and *Bifidobacterium* species. This promotes a healthier gut microbiome, improves gut barrier integrity, and reduces pathogen colonization and shedding. Species such as *Palmaria palmata* and *Ulva rigida* also supply bioactive components that improve intestinal morphology, immune function, and overall animal resilience, contributing directly to reduced pathogen contamination in meat [27,41].

### 4.3. Reduction of Contaminants

Algae demonstrate significant potential in reducing contaminants like heavy metals and environmental toxins, further enhancing meat safety. Brown seaweeds (e.g., *Fucus vesiculosus* and *Laminaria digitata*) produce polysaccharides (alginate and fucoidan) that bind heavy metals such as arsenic, lead, and mercury, minimizing their bioavailability and accumulation in animal tissues [33,35,42]. These compounds effectively sequester contaminants within the gastrointestinal tract, preventing their systemic absorption and safeguarding consumer health by reducing contaminant residues in meat products.

### 4.4. Synergistic Effects with Other Feed Additives

The efficacy of microalgae and seaweeds in promoting meat safety can be enhanced through synergistic combinations with other natural feed additives. Co-supplementation of seaweed extracts with prebiotics (e.g., galacto-oligosaccharides, GOS) or plant-derived antimicrobials (e.g., caprylic acid, thymol, and eugenol) further reduces pathogen loads. For example, combining seaweed extracts and GOS significantly decreases *Salmonella typhimurium* colonization in pigs, simultaneously enhancing gut microbiota and pathogen inhibition [43,44]. Such integrative strategies offer multifaceted mechanisms to comprehensively address pathogen control and meat safety.

### 4.5. Mechanisms of Enhancing Meat Quality

Algae-derived nutrients and bioactive compounds improve meat quality through various interconnected biochemical and physiological mechanisms. Antioxidant compounds (e.g., carotenoids, phenolic acids, phycobiliproteins) reduce lipid oxidation, maintaining meat freshness and extending shelf life [8]. Furthermore, algae-derived omega-3 fatty acids and bioactive peptides modulate immune responses and mitigate inflammatory processes in animals, enhancing meat tenderness and nutritional profile [45]. Improved gut health, achieved through prebiotic actions, indirectly contributes to meat safety by reducing pathogen loads and inflammation-related quality deterioration [46]. Collectively, these mechanisms ensure safer, higher-quality meat products.

### 4.6. Sustainability and Consumer Demand

The integration of microalgae and seaweeds into monogastric diets addresses both sustainability and consumer preferences for natural food production. By reducing reliance on synthetic antibiotics and traditional feed additives, algae-based approaches support eco-friendly livestock production, minimizing environmental impacts and aligning with consumer demand for safer, sustainably produced meat. Overall, incorporating microalgae and seaweeds into animal diets represents a comprehensive, innovative strategy for enhancing meat safety, nutritional value, sustainability, and consumer acceptance of meat products.

Table 2 summarizes the impact of various microalgae and seaweeds on meat safety traits. It highlights key bioactive compounds, the specific safety traits they influence and their mechanisms of action.

## 5. Contribution of Dietary Algae to Sustainability

Integrating algae into monogastric animal diets offers substantial benefits for enhancing sustainability in livestock production. These marine resources help mitigate greenhouse gas emissions, reduce nutrient pollution, support circular bioeconomy practices, and align with consumer preferences for environmentally friendly and ethically produced meat. Their cultivation methods and nutrient profiles make them promising alternatives to traditional feed sources, contributing to more sustainable and efficient production systems.

### 5.1. Environmental Benefits of Integrating Microalgae and Seaweeds in Animal Diets

Microalgae and seaweeds have significant potential to enhance the sustainability of livestock production by reducing greenhouse gas (GHG) emissions and improving nutrient cycling. Emerging research provides compelling evidence that incorporating specific microalgae, such as *Dunaliella salina* and *Asparagopsis taxiformis*, into animal diets can substantially decrease methane production during digestion. For instance, *Asparagopsis taxiformis* has been shown to reduce methane emissions in ruminants by up to 80%, primarily through the inhibition of methane-producing archaea in the rumen [54]. This reduction in methane, a potent greenhouse gas with a global warming potential approximately 28 times greater than carbon dioxide over a 100-year period, significantly lowers the overall carbon footprint of meat production.

Furthermore, microalgae cultivation contributes to carbon sequestration, making it a carbon-negative process. Through photosynthesis, microalgae such as *Chlorella vulgaris* and *Nannochloropsis* capture atmospheric CO₂ and convert it into biomass, which can then be incorporated into animal feed [8]. This dual role of reducing emissions and sequestering carbon underscores the potential of algae-based feed to support climate change mitigation efforts.

Microalgae and seaweeds also enhance nutrient utilization and mitigate nutrient pollution in livestock systems. Seaweeds such as *Ascophyllum nodosum* and *Ulva* improve feed efficiency by enhancing nutrient absorption in animals, thereby reducing the excretion of nitrogen and phosphorus in manure [55]. Reduced nutrient excretion helps minimize ammonia emissions and lowers the risk of nutrient runoff into waterways, which is a major driver of eutrophication and aquatic ecosystem degradation. Additionally, algae cultivation systems serve as effective bioremediation tools, absorbing excess nutrients from agricultural waste streams and wastewater, thus reducing pollution and supporting a circular economy [56].

Integrating microalgae and seaweeds into animal diets not only improves feed conversion efficiency and reduces environmental impacts but also aligns with broader sustainability goals by promoting a more circular and resource-efficient livestock production system. Future research should focus on quantifying the long-term effects of algae-based feed on methane reduction, nutrient cycling, and overall livestock productivity to validate these promising findings and support large-scale adoption in the agricultural sector.

### 5.2. Role in Circular Bioeconomy and Nutrient Recycling

Microalgae and seaweeds play a crucial role in promoting a circular bioeconomy by recycling nutrients and converting waste into valuable biomass. Microalgae cultivation systems can utilize agricultural wastewater and other nutrient-rich effluents as growth media, producing protein-rich biomass as a sustainable feed alternative [57,58]. This process reduces the need for synthetic fertilizers and imported feed ingredients, such as soybean meal, while preventing nutrient loss and environmental pollution.

In integrated multitrophic aquaculture (IMTA) systems, seaweeds are cultivated alongside fish or shellfish. In these systems, seaweeds absorb excess nutrients from fish farming operations, such as nitrogen and phosphorus, promoting efficient resource use and minimizing waste [8]. This approach not only supports sustainable aquaculture but also provides a steady supply of nutrient-rich seaweed for livestock feed. By adopting these circular bioeconomy practices, livestock producers can create more sustainable, closed-loop systems that reduce waste and optimize resource utilization.

### 5.3. Consumer and Market Perspectives on Sustainable Meat Production

Consumer demand for sustainable and ethically produced meat is driving interest in feed additives derived from microalgae and seaweeds. As awareness of environmental issues grows, consumers are increasingly seeking meat products that are produced using eco-friendly and transparent practices. Microalgae and seaweeds offer a natural and sustainable alternative to traditional feed ingredients, aligning with these market trends [39,59].

Meat enriched with omega-3 fatty acids and other bioactive compounds from microalgae and seaweeds is perceived as healthier and more nutritious. This appeals to health-conscious consumers who value the added benefits of functional foods [60]. Additionally, the use of sustainable feed sources enhances the brand image of livestock producers and meat suppliers, giving them a competitive edge in the market. By incorporating microalgae and seaweeds into animal diets, producers can meet consumer expectations for sustainability, quality, and transparency, thereby increasing market acceptance and demand for their products.

### 5.4. Practical Implications for Sustainable Livestock Production

The practical integration of algae into monogastric diets addresses key sustainability challenges in modern livestock production. These feed additives provide a means to reduce the environmental impact of meat production, including lowering greenhouse gas emissions and nutrient pollution. Their ability to grow on non-arable land with minimal freshwater requirements makes them a viable solution in regions where agricultural resources are limited. By supporting circular bioeconomy practices and aligning with consumer preferences for sustainable food, microalgae and seaweeds offer a holistic approach to improving the sustainability of livestock farming.

The sustainability benefits of incorporating microalgae and seaweeds in monogastric animal diets are diverse and impactful, addressing key environmental challenges such as greenhouse gas emissions, nutrient pollution, and resource efficiency (Table 3).

## 6. Challenges and Future Perspectives

The integration of algae into monogastric animal diets offers substantial benefits for improving meat quality, safety, and sustainability. However, several challenges hinder their widespread adoption. Addressing these challenges and exploring future perspectives will be essential for realizing their full potential.

### 6.1. Challenges and Limitations

The high production costs of microalgae and seaweeds are a significant barrier. Cultivation methods such as photobioreactors and open ponds demand substantial investments in infrastructure, energy, and water resources [62,63]. Despite technological advancements, the cost of algae-derived products remains higher than traditional feed ingredients like soybean meal. Similarly, seaweed production faces challenges related to scalability and environmental variability. Integrated multitrophic aquaculture (IMTA) systems, though promising, are still in the developmental stages and require further optimization to reduce costs and enhance feasibility.

The nutritional and bioactive composition of microalgae and seaweeds varies widely depending on species, growth conditions, and processing methods [64]. This inconsistency can affect their efficacy as feed additives. Standardizing cultivation practices and implementing stringent quality control measures are essential to ensure consistent product performance.

Introducing novel feed additives like algae faces complex regulatory challenges [8]. Approval processes often require extensive safety and efficacy evaluations, which can be time-consuming and costly. Additionally, concerns over contaminants such as heavy metals in seaweeds necessitate rigorous quality assurance protocols. Harmonizing regulatory frameworks across regions will facilitate the smoother adoption of these feed additives.

High inclusion levels of microalgae and seaweeds in animal diets can adversely affect meat’s sensory properties, such as flavor, odor, and texture [65]. Optimizing inclusion rates and employing encapsulation technologies or blending strategies can help mitigate undesirable sensory effects.

### 6.2. Future Perspectives

Innovations in cultivation systems, such as advanced photobioreactors, are crucial for improving production efficiency. Emerging techniques like hydrodynamic cavitation and supercritical fluid extraction enhance the yield and purity of valuable compounds, reducing production costs [66,67]. Genetic engineering tools, such as CRISPR/Cas9, can be employed to develop algae strains with superior growth rates and bioactive compound profiles [68].

Identifying new species of microalgae and seaweeds with higher bioactive compound content can broaden their applications [69]. For instance, *thraustochytrids* are rich in DHA, while seaweeds like *Codium* and *Pyropia* offer unique polysaccharides and antioxidants that could benefit livestock diets.

Comprehensive research on the long-term effects of algae-based feeds is necessary to evaluate their impact on animal health, growth performance and meat quality [2]. These studies will refine optimal inclusion rates and address potential challenges such as nutrient bioavailability and digestibility.

Developing economical and species-specific feed formulations is critical. Co-culturing algae with bacteria or yeast and utilizing agricultural wastewater as a nutrient source can reduce production costs. Tailored formulations that balance nutritional benefits with the sensory attributes of meat will enhance market acceptance.

Incorporating sustainability metrics into the evaluation of algae-based feeds will underscore their environmental benefits [70]. Metrics such as carbon footprint reduction, nutrient recycling efficiency and water usage will support their adoption as sustainable livestock solutions.

By addressing these challenges and pursuing innovative approaches, the integration of microalgae and seaweeds into monogastric diets can significantly enhance meat production’s quality, safety, and sustainability while aligning with global goals for sustainable agriculture.

## 7. Conclusions

Integrating algae into monogastric feeding systems offers a sustainable way to enhance meat quality, safety, and environmental sustainability in livestock production. Rich in bioactive compounds such as omega-3 fatty acids, antioxidants and polysaccharides, these marine-derived additives provide numerous benefits for animal health and meat production. By increasing omega-3 PUFA content and depositing beneficial bioactive compounds, these additives improve meat’s nutritional profile and sensory attributes like color, flavor, and texture while also extending shelf life by reducing spoilage through oxidation prevention. Additionally, their antimicrobial properties and ability to improve gut health reduce foodborne pathogens and contamination risks, ensuring safer meat products. The complementary composition of microalgae and seaweeds makes their blend promising ingredients to be included in monogastric diets.

From an environmental perspective, algae help address greenhouse gas emissions, nutrient pollution, and resource efficiency. Their cultivation requires minimal freshwater and arable land, positioning them as a sustainable alternative to traditional feed ingredients while supporting circular bioeconomy practices through nutrient recycling.

Challenges remain, including high production costs, scalability, variability in composition and regulatory hurdles. Potential impacts on meat sensory qualities, such as off-flavors at high inclusion rates, must also be carefully managed with optimized feeding strategies and processing techniques. To fully unlock their potential, research should focus on reducing production costs, identifying high-value species, and conducting long-term studies on their effects on monogastric diets. Developing cost-effective, tailored formulations and integrating sustainability metrics will be key to ensuring these feed additives are practical and impactful in commercial settings.

## Figures and Tables

**Table 1:** Effects of algae on meat quality parameters.

Feed Additive	Bioactive Compounds	Meat Quality Trait	Beneficial Effect	Main Reference
Microalgae				
*Chlorella vulgaris*	Omega-3 (ALA), carotenoids, proteins	Nutritional profile	Improves omega-3 content and antioxidant capacity	[38]
*Chlorella vulgaris*	Carotenoids, chlorophylls	Color stability	Enhances color uniformity and brightness	[39]
*Limnospira platensis*	Proteins, phycobiliproteins, β-carotene	Nutritional profile	Increases omega-3 fatty acid deposition in muscle tissue	[13]
*Limnospira platensis*	Phycobiliproteins, β-carotene	Color stability	Enhances redness and delays myoglobin oxidation	[13]
*Nannochloropsis*	Omega-3 (EPA, DHA)	Nutritional profile	Increases EPA and DHA levels in meat	[16]
*Nannochloropsis*	Lipids, antioxidants	Shelf Life	Reduces lipid oxidation, extending shelf life	[38]
Seaweeds				
*Ascophyllum nodosum*	Fucoidan, polyphenols, minerals	Eating quality	Enhances tenderness and flavor profile	[8]
*Ascophyllum nodosum*	Polyphenols, antioxidants	Shelf life	Inhibits lipid oxidation, improving shelf life	[8]
*Gracilaria*	Proteins, agar, fucoidan	Nutritional profile	Increases protein content and antioxidant capacity	[20]
*Gracilaria*	Polyphenols, dietary fiber	Shelf life	Reduces lipid and protein oxidation during storage	[20]
*Ulva*	Ulvan, phenolic compounds	Color stability	Maintains meat color by reducing oxidative damage	[8]
*Ulva*	Polysaccharides, antioxidants	Shelf life	Extends shelf life by reducing lipid oxidation	[20]

**Table 2:** Effects of algae on meat safety traits.

Feed Additive	Bioactive Compounds	Meat Safety Trait	Beneficial Effect	Mechanism of Action	Main Reference
Microalgae					
*Chlorella vulgaris*	Carotenoids, chlorophylls	Pathogen reduction	Reduces *Salmonella* and *E. coli* in meat	Disrupts bacterial membranes and reduces colonization	[38]
*Limnospira platensis*	Phycocyanin, phycobiliproteins	Antimicrobial activity	Inhibits *Listeria monocytogenes* and *Staphylococcus aureus*	Binds to bacterial membranes and disrupts cell function	[47]
*Nannochloropsis*	EPA, DHA, polyphenols	Pathogen inhibition	Decreases *Campylobacter jejuni* and *Salmonella enteritidis*	Inhibits bacterial growth and biofilm formation	[48]
*Tetraselmis*	Polysaccharides, antioxidants	Toxin reduction	Lowers mycotoxin levels in meat	Binds to mycotoxins and reduces bioavailability	[49]
Seaweeds					
*Ascophyllum nodosum*	Fucoidan, polyphenols	Pathogen reduction	Reduces *E. coli*, *Salmonella* and *Listeria* spp.	Disrupts bacterial membranes and inhibits growth	[50]
*Fucus vesiculosus*	Polysaccharides, antioxidants	Heavy metal detoxification	Reduces accumulation of lead, arsenic, and mercury	Binds to heavy metals and reduces their bioavailability	[51]
*Palmaria palmata*	Laminarin, fucoidan	Improved gut health	Reduces pathogen shedding and improves microbial balance	Prebiotic effect promoting beneficial bacteria	[41]
*Gracilaria*	Agar, polyphenols	Pathogen inhibition	Inhibits *Staphylococcus aureus* and *E. coli*	Reduces bacterial growth and biofilm formation	[52]
*Ulva lactuca*	Ulvan, phenolic compounds	Toxin reduction	Reduces bacterial toxins and contamination risk	Inhibits toxin production and bacterial growth	[22]
*Sargassum*	Fucoidan, polyphenols	Pathogen reduction	Reduces *Clostridium perfringens* and *E. coli*	Inhibits spore germination and bacterial proliferation	[53]

**Table 3:** Sustainability benefits of algae in livestock production.

Feed Additive	Sustainability Benefit	Mechanism of Action	Type of Impact	Main Reference
Microalgae				
*Chlorella vulgaris*	Reduction in greenhouse gas emissions	Decreases methane production during digestion	Lowers the environmental footprint of livestock	[61]
*Limnospira platensis*	Minimal land and water use	Cultivated on non-arable land with low water requirements	Reduces pressure on agricultural resources	[2]
*Nannochloropsis*	Carbon sequestration	Captures CO_2_ during cultivation	Supports climate change mitigation	[16]
*Tetraselmis*	Bioremediation of wastewater	Removes excess nutrients from agricultural runoff	Reduces nutrient pollution and eutrophication	[57]
Seaweeds				
*Ascophyllum nodosum*	Nutrient recycling	Uses wastewater for cultivation	Improves resource efficiency and reduces pollution	[8]
*Fucus vesiculosus*	Heavy metal sequestration	Binds and removes heavy metals from water	Reduces contamination in marine environments	[51]
*Gracilaria*	Supports circular bioeconomy	Converts waste nutrients into biomass	Promotes sustainable nutrient recycling	[20]
*Palmaria palmata*	Reduction in nitrogen runoff	Enhances feed efficiency, reducing nitrogen excretion	Lowers nutrient pollution in soil and water	[8]
*Sargassum*	Climate change mitigation	Reduces methane emissions in animal digestion	Decreases overall greenhouse gas emissions	[20]
*Ulva lactuca*	Integrated Multitrophic Aquaculture	Recycles nutrients from fish farming	Enhances resource efficiency and reduces waste	[8]

## Data Availability

No new data were created or analysed in this study. Data sharing is not applicable to this article.
